# Accessibility Analysis of Worldwide COVID-19-Related Information Portals

**DOI:** 10.3390/ijerph191912102

**Published:** 2022-09-24

**Authors:** Patricia Acosta-Vargas, Sylvia Novillo-Villegas, Belén Salvador-Acosta, Manuel Calvopina, Nikolaos Kyriakidis, Esteban Ortiz-Prado, Luis Salvador-Ullauri

**Affiliations:** 1 Intelligent and Interactive Systems Laboratory, Universidad de Las Américas, Quito 170125, Ecuador; 2 Carrera de Ingeniería en Producción Industrial, Facultad de Ingeniería y Ciencias Aplicadas, Universidad de Las Américas, Quito 170125, Ecuador; 3 Facultad de Tecnologías de Información, Universidad Latina de Costa Rica, San José 11501, Costa Rica; 4 Facultad de Medicina, Universidad de Las Américas, Quito 170125, Ecuador; 5 One Health Research Group, Universidad de las Américas, Quito 170125, Ecuador; 6 Department of Software and Computing Systems, University of Alicante, 03690 Alicante, Spain

**Keywords:** accessibility, analysis, COVID-19, websites, web content accessibility guidelines 2.1

## Abstract

Since the beginning of the COVID-19 pandemic, communication technology has demonstrated its usefulness in sharing and receiving health data and communicating with the public. This study evaluated the accessibility of 199 websites containing official COVID-19 information related to medical schools, governments, ministries, and medical associations, obtained from the Geneva Foundation for Medical Education and Research website. We used the Web Content Accessibility Guidelines 2.1 to evaluate web accessibility, using a six-phase process with an automatic review tool. The study results reveal that the highest number of barriers encountered are concentrated in the perceivable principle with 6388 errors (77.8%), followed by operability with 1457 (17.7%), then robustness with 291 (3.5%), and finally understandability with 78 errors (0.9%). This study concludes that most COVID-19-related websites that provide information on the context of the pandemic do not have an adequate level of accessibility. This study can contribute as a guide for designing inclusive websites; web accessibility should be reviewed periodically due to technological advances and the need to adapt to these changes.

## 1. Introduction

World Health Organization (WHO) reports reveal that 15% of the world’s population has a disability [1]. The prevalence of people with disabilities is significantly higher in developing countries [2]. As COVID-19 has a global large-scale impact [3], it is essential to recognize that accessibility is fundamental to protecting the most vulnerable people whose disabilities make it difficult to access other health and educational services, including schooling, transportation, and health-related information.

During the COVID-19 pandemic, technology has enabled governments and health-related institutions to inform and share content intended to increase health promotion [4]. In this sense, several websites were created to report COVID-19 epidemiological data, prevention strategies, and other measures to try to ameliorate the impact of the pandemic worldwide [5]. These websites have become a powerful tool for the public to obtain information and to use as contrast with misinformation, i.e., information that was not generated by governments, including countries’ health departments and non-governmental organizations, as well as universities or medical centers.

However, not all websites are accessible to all citizens, especially those whose eyesight has diminished with advancing age due to presbyopia. Web-based communication is essential, so removing accessibility barriers is required for websites to serve all people who rely on assistive technologies, such as screen readers and captioning services, to use various web-based resources, documents, or applications [6].

WHO reports [7] that about 80% of cases of low vision and about 70% of cases of blindness can be prevented. The main clinical criterion in visual impairment is the visual acuity in the better eye, which shows the degree of deficiency, impairment, or deficit in visual function.

One of the benefits of accessibility in websites is to increase the number of people accessing the content and to keep the current users loyal, as they remain using the applications after having obtained a satisfactory experience. In addition, an accessible website enhances an organization’s image and demonstrates its social responsibility to the public.

In some countries, designing for web accessibility is a legal obligation, so the World Wide Web Consortium has created a standard [8] for designing accessible websites, called the Web Content Accessibility Guidelines (WCAG) 2.1. These guidelines aim to provide [9] “a single, shared standard for web content accessibility and how to make web content more accessible to people with disabilities”.

Web accessibility [4] means that people with disabilities can use the Web, referring to a design that allows people with disabilities to perceive, understand, navigate, and interact with the Web while providing the content. Accessibility benefits others, including the elderly, whose abilities are reduced due to age.

The standard is classified into three levels, level A, the most basic; level AA, intermediate; and level AAA, the most rigorous, and four accessibility principles [4], such as being perceivable, operable, understandable, and robust.

Perceivable [4] refers to the content as a website interface that all its users must perceive. They must have perceptible alternatives for blind or deaf people and tell them that the audio description of the videos, their titles, and the sign language of the content is accessible. Color is also part of the group of relevant indicators because a percentage of the population has a condition that cannot be perceived correctly.

According to the World Wide Web Consortium (W3C) rules, the operable principle [4] ensures that all the website’s functionality is based on the keyboard. Because most people are used to navigating websites with a mouse, navigation becomes almost impossible if the mouse fails or a group of the population cannot use it for various reasons. The times in which the information also changes the influence; thus, they must be sufficiently studied so that all potential users, regardless of their condition or age, can understand them. On the other hand, it is important not to design content or tools that can cause seizures: there are web design tools that are fascinating, which quickly change shapes and colors, and that provide a website a considerable impact, but many of them can cause attacks or epileptic seizures, which can be prevented.

An understandable [4] website refers to both the form and the background of the texts of a website. The latter must use a font that all users can read. Concerning the substance, the correct use of abbreviations, idioms, and neologisms should be contemplated, so that all users understand them.

The robust principle [4] means that websites or applications must be compatible with all web browsers, operating systems, devices, and assistive technology applications, such as screen readers for the blind, including JAWS, NVDA, talkback, and VoiceOver.

In this context, our research question focuses on whether official websites providing information about COVID-19 are accessible and inclusive.

Accessibility means easily accessing a website for any user [6]. This accessibility is related to all potential users and not only to people with hearing, visual, physical, cognitive, and communication disabilities; it also includes older adults who require it.

For this reason, official websites with COVID-19 information should be accessible to all users in order to implement timely prevention strategies from the first level of care and biosecurity measures to control the COVID-19 pandemic according to international standards. Unfortunately, when a website is not accessible, users abandon the website and seek information on social networks or other misinformation channels that attempt to mislead, confuse, and alarm the public.

This research evaluates 199 websites related to COVID-19 information based on Website Accessibility Conformance Evaluation Methodology (WCAG-EM) 1.0 [10] versus WCAG 2.1.

The applied method comprises an automatic review using the WAVE [11] in six stages detailed in the Methods Section. This research can help future research associated with accessibility for any website. In addition, it can be an input to help application developers to design software with artificial intelligence to help experts evaluate accessibility in websites applying WCAG 2.1.

The rest of the manuscript is structured as follows: Section 2 presents the background and related works; Section 3 shows the methods and materials; Section 4 contains the results and analysis; Section 5 contains the discussion of the evaluated websites; and finally, Section 6 includes the conclusions, limitations, and future works obtained in this research.

## 2. Background and Related Works

The authors of [4] noted that, during the COVID-19 pandemic, technology was essential for health agencies to communicate with the public. The study analyzed two COVID-19 government websites using the WCAG standard. The analyzed websites required improvement in the accessibility of COVID-19 information as it plays a crucial role in the health and safety of the public.

The exploratory study of [12] investigated the web accessibility of 54 official COVID-19 vaccine registry websites in the US and their compliance with WCAG 2.0 and 2.1 guidelines. The results revealed compliance with WCAG 2.0 and 2.1 guidelines. Non-compliance creates difficulties for users with disabilities to access information on websites and the article proposed recommendations for governments to improve the accessibility of their websites.

The study of [13] argued that the United States Centers for Disease Control and Prevention allocates vaccines to individual states for distribution. Individuals seeking COVID-19 vaccine eligibility require information and thus need to visit health department websites. However, different levels of technological expertise, reading skills, and language preferences may prevent individuals from obtaining the necessary information regarding COVID-19. The results indicate that 14% of the websites did not have complete smartphone visibility and 18% were in English. The authors suggested that websites should enable mobile access to information in multiple languages, using a language that is easier for the public to understand to address these problems.

The research of [14,15] addressed the issue by considering the situation of the health crisis that required greater attention and reliance on trustworthy sources for citizens, especially those organizations representing competent authorities, such as the World Health Organization (WHO).

Therefore, information must be understandable by and accessible to all people, regardless of technology access, language, culture, or disability, according to the W3C [4]. The study of [14] contributed to the development of proposals and suggested ways to improve the accessibility of healthcare content, especially vulnerable in this pandemic. The results revealed that understandable and perceptible principles are essential for communicating with citizens. Additionally, they are directly related to easy-to-understand content in the first case and offer text alternatives for non-textual content in the second case, especially for visually impaired people who use readers and even older people who have vision problems due to aging.

According to WHO [7] figures worldwide, it is estimated that approximately 1.3 billion people live with some form of visual impairment. The leading causes of poor vision are uncorrected refractive errors and cataracts. Most people with poor vision are over 50 years of age; however, vision loss can affect people of all ages. Vision impairment poses a substantial global economic burden, as the annual costs due to lost productivity associated with vision impairment from uncorrected myopia and presbyopia are estimated to be USD 244 billion and USD 25.4 billion, respectively, worldwide. According to several studies, visual impairment is associated with decreased quality of life [1,2,6,7], limitations in the performance of daily activities, and increased risk of accidents, depression, social isolation, and other alterations in health status [16].

In general, Web accessibility means that people with disabilities can use the Web, and it refers to a Web design that allows people to perceive, understand, navigate, and interact with the Web while providing the content [17]. In addition, Web accessibility benefits everyone, including the elderly who have had their abilities reduced because of age. The W3C [18] Web Accessibility Initiative (WAI) is an organization that introduced accessibility guidelines known as Web Content Accessibility Guidelines (WCAG). It provides WCAG 1.0 [19], WCAG 2.0 [20], WCAG 2.1 [8], WCAG 2.2 [21], and WCAG 3.0 [22] for three levels of conformance A, AA, and AAA. WCAG aims to ensure that websites or their content are accessible and inclusive for people with disabilities.

According to World Bank statistics, one billion people worldwide, 15% of the world’s population [1], live with some form of disability, such as visual impairment, hearing impairment, physical disability, or cognitive or neurological impairment.

According to World Health Organization (WHO) reports, among the entire population of the world, 3.2% of people have a visual impairment, 6% of people have hearing or hearing difficulties, 2.6% of people have a neurological difficulty, and 1% of people have physical disabilities or require a wheelchair [23].

From the described above, our research addresses and analyzes websites containing information on COVID-19 to share the results found in the evaluation of the total sample of sites obtained from Geneva Foundation for Medical Education and Research [24]; in addition, WHO is included.

## 3. Materials and Methods

The evaluation process comprised two phases: (1) selecting the automatic evaluation tool that meets the accessibility standards and (2) evaluating the accessibility of the websites with the tool that achieves the best results in the selection.

Phase 1: Among the tools most commonly used by accessibility experts [6,11,15,17,25], we found AccessMonitor, Achecker, eXaminator, TAW, Tenon, WAVE, Web Accessibility Checker, and Mauve++ Tool.

In the evaluation, we considered some parameters such as the accessibility four principles (perceivable, operable, understandable, and robust), the application of the guidelines, and the version and the level of accessibility (A, AA, and AAA). In addition, we analyzed features such as loading times, license, report, plugin, support and maintenance, functionality, portability, usability, connectivity, and security. To select the most appropriate tool, we present a summary in Table 1, containing ID, tool, WCAG 2.1 and 2.0, level A, AA, AAA, and total and percentage score.

The results indicate that those that meet 100% of the parameters of the tools evaluated correspond to Tenon, WAVE [11,25], Web Accessibility Checker, and Mauve++ Tool. In the selection analysis of the auto-math evaluation tool, parameters such as loading times, license, report, plugin, support, functionality, portability, usability, connectivity, and security were considered. In the evaluation, 1 was recorded if the tool complied, and 0 if it did not comply. Similarly, the eight tools were then calculated for compliance or non-compliance with the level of accessibility, and the percentage score was calculated. It was observed that the tools Tenon, WAVE, Web Accessibility Checker, and Mauve++ Tool reach 13.9% each.

In addition, we added criteria for analyzing the quality of the tool. Although these tools are an invaluable aid in evaluating the accessibility of websites, readers should understand that they are far from infallible and have limitations that can lead to false positives. The evaluation of the tools depends on the experience of the evaluators as well as the evaluators’ judgments.

The results are summarized in Table 2, containing the identifier, loading times, license, report, plugin, support, functionality, portability, usability, connectivity, security, total, percentage additional score, and percentage average.

To calculate the percentage average, we used the percentage score from Table 1 with the additional percentage score. As a result, the tool that has the best performance in the evaluation is WAVE with 17.3%, followed by Mauve++ Tool with 16.1% and the third-place Tenon with 13.7%.

**Figure 1:**
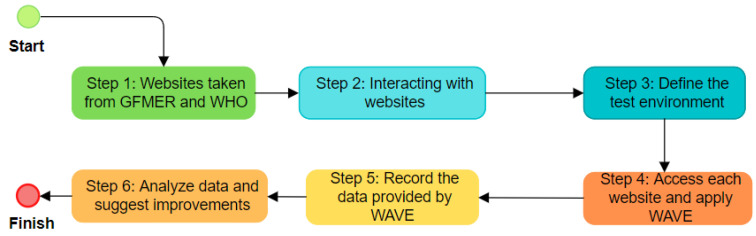
Automatic review method applied to the websites.

Phase 2: Once the tool was defined, the sample of websites to be evaluated was obtained from the Geneva Foundation for Medical Education and Research (GFMER) [24]. The data were available in the Mendeley repository. A total of 198 GFMER websites were evaluated, and the WHO website was also included. The evaluation began on 15 October 2021, and five evaluators participated. An automatic review method was applied using the WAVE tool for the evaluation. The method is described in six steps, summarized in Figure 1.

In step 1, we took the total sample of GFMER [24] websites and included one of the most consulted websites in pandemics, such as the World Health Organization (WHO). We selected 199 websites to evaluate accessibility. Table 3 presents a sample of the first 40 websites evaluated; the total evaluated websites and the analysis are available with open access to the data in the Mendeley Data repository [25].

In step 2, we experimented with the navigation and interaction with each website that provides official information about COVID-19. In step 3, we defined the test environment so that the same functions are performed on all websites to be evaluated. The test environment is used for user acceptance testing activities. In this phase, we previously installed the Google Chrome browser version 101.0.4951.64 for 64 bits, added the WAVE Evaluation Tool extension under, and offered by WebAIM, version 3.1.6, with an updated date of 14 October 2021, under the Windows 10 operating system. Then, we performed the following actions: (1) open the browser; (2) type the URL address of the website; and (3) load the main page.

In step 4, once the actions of step 3 are performed, we run the WAVE plugin; we waited for the website analysis to load. We reviewed the summary and detailed information from the WAVE tool.

In step 5, we recorded the data from the Summary section in a spreadsheet; in this study, we used Microsoft Excel 365. We examined the values of Errors, Contrast Errors, Alerts, Features, Structural Elements, and ARIA. In addition, we inspected each of the barriers related to Errors and Contrast Errors from the Details option. The data were classified according to the four accessibility principles and the success criteria.

Finally, in step 6, we analyzed the data and identified the websites with the highest number of errors and contrast errors that should be corrected to improve accessibility. For this study, we considered level AA. The results of this analysis are detailed in the Discussion section.

## 4. Results and Analysis

This section presents the evaluation results of the total GFMER sample, including 199 websites; they were classified and tabulated according to the four accessibility principles of WCAG 2.1 [8]. We considered the principle of being perceivable related to users with visual impairment, specifically low vision, whose visual ability decreased due to presbyopia related to aging. The information was summarized using Microsoft Excel with the support of dynamic reports. Table 4 presents the evaluation of the first 40 websites; the total sample is in the open data repository Mendeley [25].

In Table 4, errors [11] indicate a significant accessibility problem; the number of errors is a good indicator of real end-user accessibility problems that require urgent resolution. The contrast errors [11] parameter reveals the contrast and use of color vital for accessibility; visually impaired users must be able to perceive the page content.

Alerts [11] correspond to elements that may cause accessibility problems, where the evaluator is the one who decides the accessibility impact of the website.

Features [11] imply that the elements can improve accessibility when implemented correctly. Structural elements are related to some web page title, indicating that it has been marked as a top-level title or various milestones.

Finally, accessible rich internet applications (ARIA) [11] is a collection of attributes that define how to perform web content and applications; thus, it influences accessibility when misused.

**Figure 2:**
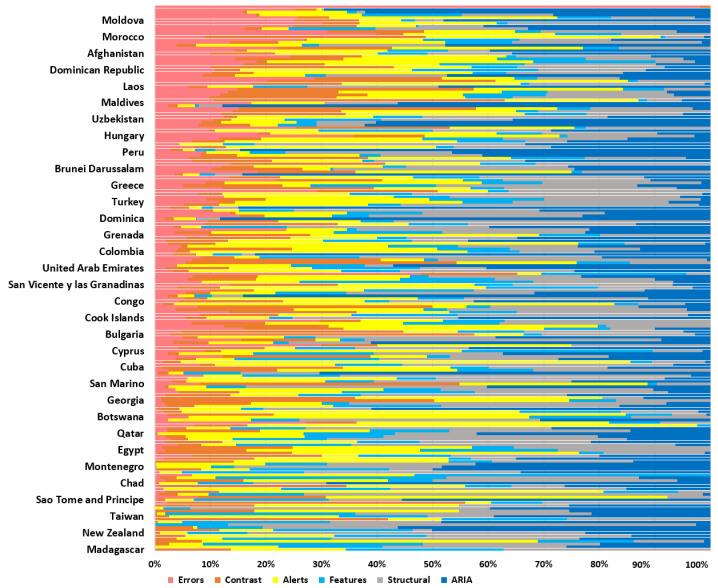
Summary of 199 websites and variables analyzed with WAVE.

Figure 2 shows errors in pink, contrast errors in orange, alerts in yellow, features in light blue, structural errors in gray, and ARIA errors in blue. The results show Madagascar, Lithuania, United States of America, Italy, Latvia, Latvia, Barbados, New Zealand, Central African Republic, Slovenia, Iran, Sweden, Cayman Islands, Taiwan, United Kingdom, Timor-Leste, Denmark, and Turkmenistan have zero accessibility errors. Likewise, Lithuania, Kazakhstan, France, Sri Lanka, Iran, Botswana, Maldives, Denmark, Montenegro, El Salvador, Norway, Australia, Poland, Ecuador, Qatar, Croatia, Samoa, Ethiopia, and South Africa have zero contrast errors. The information reveals that the countries mentioned above meet an acceptable level of accessibility. On the other hand, Syria and the Russian Federation present a high number of errors, totaling 41.81%; likewise, Georgia, the Russian Federation, Serbia, and Morocco show a total of 16.3% in contrast errors. These results show that the websites of the mentioned countries should urgently correct the websites to reduce accessibility barriers, especially for users with a low vision directly related to contrast errors.

The results reveal that the leading websites with significant accessibility problems do not necessarily correspond to some developed or developing countries; it is supported by the study of the million made by WAVE [11], where the percentage of low contrast errors corresponds to 86.3%, mainly related to the perceivable principle.

Table 5 presents the four accessibility principles according to WCAG 2.1, the guideline, the success criteria, the level of accessibility, and the number of barriers identified in the analyzed websites, all related to the corresponding principle.

The perceivable principle [8] is related to users with low vision and corresponds to the success criterion 1.4.3 of minimum contrast; as it can be seen, it is the criterion with the most accessibility barriers with 54.6%, followed by 1.1.1 of missing alternative text with 16.6% and 2.4.4 of link purpose with 9.5%. The rest of the success criteria are under 7.1%.

**Figure 3:**
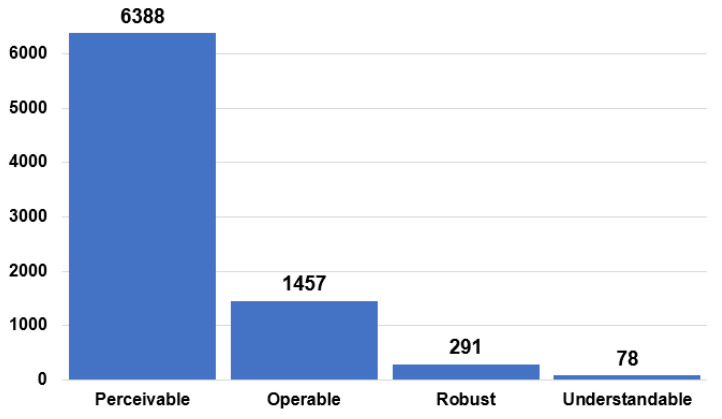
Web accessibility principles according to WCAG 2.1.

Figure 3 presents a summary of the errors identified according to the four principles of accessibility [8]. We can observe that the principle repeated most frequently in the analysis is perceivable with 6388, representing 77.8%. This principle relates to the information transmitted by non-textual content through textual alternatives. Screen readers detect the presence of images and other non-textual content, but they cannot interpret their content. For example, they will not know whether the content is a photograph of an object or a decorative figure. It is the developers’ responsibility to communicate to the screen reader, and therefore to the end user, the information or function provided by the non-textual content. When creating a textual alternative, the first thing to consider is how the page will be presented or “heard” when the images or non-textual content are not displayed. Following this is the operable principle with 17.7%, followed by robust with 3.5%, and finally understandable with 0.9%.

**Figure 4:**
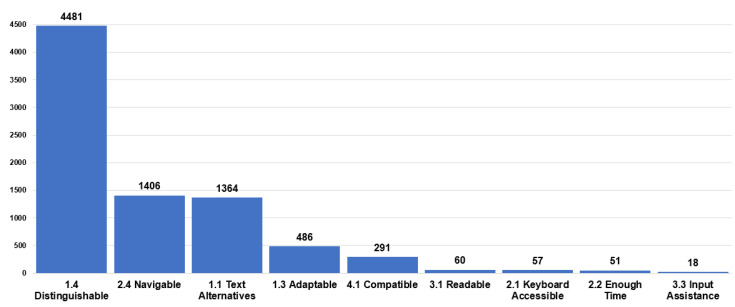
Summary of the identified accessibility guidelines.

Figure 4 presents the problems related to the accessibility guidelines that are repeated most frequently as 1.4 [8], which corresponds to distinguishable with 54.6%, followed by 2.4, which refers to navigable with 17.1%, followed by 1.1 [8] related to text alternatives with 16.6%. The rest of the problems are less than 5.9%. We can see that the most significant number of problems are related to the perceivable principle.

**Figure 5:**
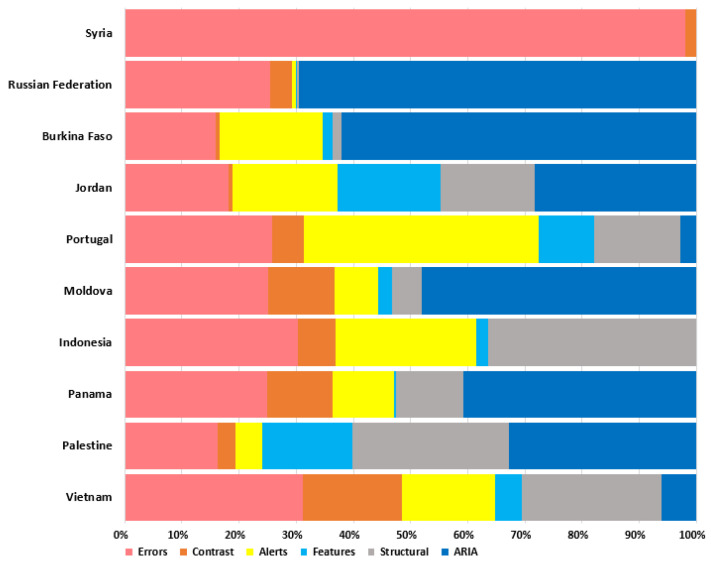
Summary of top 10 websites with the highest number of accessibility issues.

Figure 5 presents the top 10 websites with the highest number of accessibility problems. The websites with the highest number of errors corresponded to those in Syria with 34.2%, followed by the Russian Federation with 34.1%, Burkina Faso with 13.8%, and the rest of the websites with less than 5%.

## 5. Discussion

The results show the most significant number of barriers are concentrated in the perceptible principle with 77.8%, which contrasts with the study of the million applied by WAVE [11], followed by the operable principle with 17.7%, robust with 3.5%, and finally, understandable with 0.9%. The guideline with the highest number of errors corresponds to distinguishable with 54.6%, followed by navigable with 17.1% and text alternatives with 16.6%.

The success criteria with the most barriers correspond to contrast with 54.6%, followed by missing alternative text with 16.6% and link purpose with 9.5%. The level of accessibility most affected corresponds to AA with 61.6%, followed by A with 38.4%. This study suggests that the websites providing information on COVID-19 do not have an adequate level of accessibility, according to WCAG 2.1 [8].

Of the websites evaluated, we found that the countries with zero contrast errors are the Maldives, France, Ethiopia, South Africa, Kazakhstan, El Salvador, Croatia, Samoa, Ecuador, Norway, Qatar, Botswana, Australia, Poland, Montenegro, Sri Lanka, Iran, Lithuania, and Denmark.

In the analysis of the four accessibility principles in Table 5, we can observe that the most frequently repeated error is the perceivable principle related to visual impairment problems.

On the other hand, using the WAVE tool [11] as an aid does not offer a complete solution; a manual review report by a human expert is needed to complement the study. In future studies, we will apply combined and heuristic methods for manual review [6,17]. However, this tool can be used for comparative purposes to understand the behavior of a website regarding accessibility issues.

From the results obtained in this research, it can be concluded that the evaluated websites must be improved to reach an adequate level of accessibility, for which they must apply the WCAG 2.1 [8]. to achieve the minimum AA level required by the WCAG 2.1, we suggest:

Build minimalist and lightweight designs to make the website load faster; this is highly valued by users, even representing a reduction in the use of data from their devices [8].

Reduce the number of clicks to reach the content efficiently; exploring a website cannot become a journey through a maze with no way out [6,8]. Use typography that represents the essence of the brand but is also universal. Avoid justifying texts with simultaneous right and left alignment; preferably, use left alignment and write short paragraphs to avoid visual fatigue [8].

To have a quality design on the website, taking care of the graphic line of the page [18,19,20], using an appropriate color palette and contrast, an attractive structuring of the contents, and a correct distribution of white spaces.

Include alternative mechanisms [8,18] to present the same information to people with some visual disability, such as color blindness or allowing the configuration of monochromatic screens.

Place good quality images with the Alt tag or alternative text and title [6,8,17]; the tags function as pop-up descriptions and ensure accessibility to the content from any browser.

Design a simple navigation menu that can be easily located [6,8], and use short, precise words that do not generate confusion because they are similar or very technical. It is essential not to exceed the number of sections, a maximum of eight, and levels or sub-sections, a maximum of two.

Maintain a balance between quality and quantity of content for each element [6,8,17], such as images, videos, tables, and audio.

Include a space for contact information that is easy to find and always up to date; avoid including banners or moving content, such as gifs, as they can be challenging to read, and users may confuse them with unwanted advertising [6,8,11].

Apply auto-magnetic review tools, such as WAVE [11], during the website design cycle as a quick and low-cost option. Finally, it is significant to perform development evaluations early, using accessibility validators to find early web development and accessibility issues.

## 6. Conclusions, Limitations, and Future Works

The errors found in the evaluated websites were mostly related to low contrast representations, images without alternative text, empty links, entries without labels in forms, empty buttons, and documents without language attributes. Many accessibility barriers extend beyond automated reviews, requiring qualitative analysis, and user testing. Nevertheless, we can begin to reduce these types of errors on websites by applying automatic review tools that do not require much technical effort, time, or budget.

The evaluation process includes two types of problems: (1) errors that must be corrected urgently as the “errors” and (2) errors that can wait to be corrected and will depend on the criteria of an evaluator who can determine if errors should be corrected regarding specific contrast errors, alerts, structural errors, ARIA for the website to meet the minimum required level of accessibility such as AA [8].

One of the limitations is that the accessibility study of the COVID-19 websites was conducted with experts in web accessibility, not with users with any disability. Future work will expand the study with tests applied to patients with visual impairment related to low vision. This study can contribute as a guide to the design of accessible and inclusive websites. On the other hand, it is essential to disseminate and review knowledge about current legislation and the bodies that regulate web accessibility. Web accessibility has to be reviewed periodically due to the technological advances that occur and the need to adapt to these changes.

Accessibility should be an essential consideration at all stages of the software development cycle, as well as quality and usability, and can be evaluated at different stages of the application development cycle to achieve this goal. The essential task should be to disseminate knowledge on how to implement web accessibility effectively and focus on innovative implementation techniques that will attract the attention of developers.

Applying WCAG 2.1 is a challenge for web and mobile application development professionals to raise awareness of this discipline, and they must be updated on accessibility guidelines to adapt web portals to the new WCAG versions.

This research presented recommendations for good practices that stimulate different actors and stressed the importance of joint efforts in the search of solutions to eliminate access barriers to COVID-19 information websites, allow advances in human development, and promote accessibility policies to improve the quality of life [1] of all people.

Finally, one of the challenges [26] of medicine is implementing health technology and the Internet of Medical Things (IoMT) to connect various medical devices that enable usable and accessible medical care. On the other hand, technology such as blockchain will help to reduce security risks for people by including systems such as hybrid technologies, combining knowledge from big data analytics, data mining, and artificial intelligence to achieve more inclusive, secure, and practical websites.

## Figures and Tables

**Table 1:** Selection of the automatic review tool.

ID	Tool	WCAG 2.1	WCAG 2.0	Level A	Level AA	Level AAA	Total	% Score
1	AccessMonitor	0	1	1	1	1	4	11.1
2	Achecker	0	1	1	1	1	4	11.1
3	eXaminator	0	1	1	1	1	4	11.1
4	TAW	0	1	1	1	1	4	11.1
5	Tenon	1	1	1	1	1	5	13.9
6	WAVE	1	1	1	1	1	5	13.9
7	Web Accessibility Checker	1	1	1	1	1	5	13.9
8	Mauve++ Tool	1	1	1	1	1	5	13.9

Where 1 = Complies; 0 = Fails.

**Table 2:** Additional parameters for tool selection.

ID	Lt	L	R	Pn	St	F	P	U	C	S	Total	% Additional Score	% Average
1	2	1	1	0	1	1	1	1	1	1	10	8.0	9.6
2	2	1	1	1	1	1	1	1	1	1	11	8.8	10.0
3	1	1	1	0	1	1	1	0	1	1	8	6.4	8.8
4	2	0	2	0	3	3	3	3	2	2	20	16.0	13.6
5	3	0	2	0	2	2	2	2	2	2	17	13.6	13.7
6	3	1	3	1	3	3	3	3	3	3	26	20.8	17.3
7	2	1	1	0	1	1	1	1	1	1	10	8.0	10.9
8	2	1	2	0	3	3	3	3	3	3	23	18.4	16.1

Where ID = identifier, Lt = loading times, L = license, R = report, Pn = plugin, St = support, F = functionality, P = portability, U = usability, C = connectivity, S = security. Excellent = 3, Very Good = 2, Good = 1, Regular = 0.

**Table 3:** Sample of the first 40 websites evaluated.

#	Country	Domain	URL
1	Afghanistan	af	https://moph.gov.af/ (accessed on 19 September 2022)
2	Albania	al	https://en.wikipedia.org/wiki/Ministry_of_Health_and_Social_Protection (accessed on 19 September 2022)
3	Algeria	dz	https://www.facebook.com/SanteDZA/ (accessed on 19 September 2022)
4	Andorra	ad	https://www.govern.ad/ministeri-de-salut
5	Angola	ao	https://www.sepe.gov.ao/ao/gov/sepe/ministerios/detalhe/20/ (accessed on 19 September 2022)
6	Anguilla	ai	http://gov.ai/ministry.php?id=2 (accessed on 19 September 2022)
7	Antigua and Barbuda	ag	https://ab.gov.ag/detail_page.php?page=29 (accessed on 19 September 2022)
8	Argentina	ar	https://www.argentina.gob.ar/salud/coronavirus-COVID-19 (accessed on 19 September 2022)
9	Armenia	am	http://www.moh.am/ (accessed on 19 September 2022)
10	Australia	au	https://www.health.gov.au/ (accessed on 19 September 2022)
11	Austria	at	https://www.sozialministerium.at/ (accessed on 19 September 2022)
12	Azerbaijan	az	http://www.health.gov.az/ (accessed on 19 September 2022)
13	Bahamas	bs	https://COVID19.gov.bs/ (accessed on 19 September 2022)
14	Bahrain	bh	https://www.moh.gov.bh/ (accessed on 19 September 2022)
15	Bangladesh	bd	http://www.mohfw.gov.bd/ (accessed on 19 September 2022)
16	Barbados	bb	https://gisbarbados.gov.bb/covid-19/ (accessed on 19 September 2022)
17	Belarus	by	http://minzdrav.gov.by/ (accessed on 19 September 2022)
18	Belgium	be	https://www.health.belgium.be/fr (accessed on 19 September 2022)
19	Belize	bz	https://www.health.gov.bz/ (accessed on 19 September 2022)
20	Benin	bj	https://sante.gouv.bj/ (accessed on 19 September 2022)
21	Bhutan	bt	http://www.moh.gov.bt/ (accessed on 19 September 2022)
22	Bolivia	bo	https://www.minsalud.gob.bo/ (accessed on 19 September 2022)
23	Bosnia and Herzegovina	ba	http://www.fbihvlada.gov.ba/english/ministarstva/zdravstvo.php (accessed on 10/5/2022)
24	Botswana	bw	https://www.moh.gov.bw/ (accessed on 19 September 2022)
25	Brazil	br	https://coronavirus.saude.gov.br/ (accessed on 19 September 2022)
26	Brunei Darussalam	bn	http://www.moh.gov.bn/ (accessed on 19 September 2022)
27	Bulgaria	bg	http://www.mh.government.bg/ (accessed on 19 September 2022)
28	Burkina Faso	bf	http://www.sante.gov.bf/ (accessed on 19 September 2022)
29	Burundi	bi	http://minisante.bi/ (accessed on 19 September 2022)
30	Cabo Verde	cv	https://www.facebook.com/ministeriodasaude.cv/ (accessed on 19 September 2022)
31	Cambodia	kh	http://moh.gov.kh/ (accessed on 19 September 2022)
32	Cameroon	cm	http://www.minsante.cm/ (accessed on 19 September 2022)
33	Canada	ca	https://www.hc-sc.gc.ca/ (accessed on 19 September 2022)
34	Cayman Islands	ky	http://www.ministryofhealth.gov.ky/ (accessed on 19 September 2022)
35	The Central African Republic	cf	https://www.sante.gouv.cf/ (accessed on 19 September 2022)
36	Chad	td	https://sante-tchad.org/ (accessed on 19 September 2022)
37	Chile	cl	https://www.minsal.cl/nuevo-coronavirus-2019-ncov/ (accessed on 19 September 2022)
38	China	cn	http://en.nhc.gov.cn/ (accessed on 19 September 2022)
39	Colombia	co	https://www.minsalud.gov.co/salud/publica/PET/Paginas/Covid-19_copia.aspx (accessed on 10 May 2022)
40	Comoros	km	https://stopcoronavirus.km/ (accessed on 19 September 2022)

**Table 4:** Sample of the first 40 websites evaluated with WAVE.

#	Domain	Errors	Contrast Errors	Alerts	Features	Structural Elements	ARIA	1.1.1	1.3.1	1.4.3	2.1.1	2.2.2	2.2.1	2.4.1	2.4.2	2.4.4	2.4.6	3.1.1	3.3.2	4.1.2
1	af	43	49	79	38	65	152	10	1	49	6			1		13		12		
2	al	4	118	151	0	25	41	2	1							3	1		1	
3	dz	23	18	18	30	36	688	13	1	18						8		1		
4	ad	8	2	4	3	10	0	2		2						6				
5	ao	7	6	2	2	24	4	3		6						4				
6	ai	1	23	2	4	13	0			23								1		
7	ag	6	2	7	0	5	0	5		2								1		
8	ar	1	3	7	42	35	13			3						1				
9	am	20	19	42	2	25	0	20		19										
10	au	1	0	14	13	28	10	1												
11	at	1	1	195	111	265	420			1						1				
12	az	47	44	73	14	35	0	32					1			13		1		
13	bs	10	19	118	50	42	8	3		19						7				
14	bh	38	34	49	36	56	23	5		34						33				
15	bd	34	69	40	3	22	0	32		69		1				1				
16	bb	0	7	34	21	47	18			7										
17	by	32	92	58	16	18	0	29		92						3				
18	be	15	3	8	17	76	53	14	1	3										
19	bz	1	3	8	0	1	0	1												
20	bj	25	3	48	53	55	74	13		3						12				
21	bt	36	26	137	21	73	55	8		26	13	1				14		1		
22	bo	54	1	42	35	33	36	54		1										
23	ba	8	12	31	1	12	0	3		12						4		1		
24	bw	3	0	30	9	7	0	2										1		
25	br	1	49	31	12	14	38	1		49										
26	bn	20	32	10	1	85	48	3	5	32						12				
27	bg	7	135	22	8	68	7	4	1	135	1					1				
28	bf	559	23	636	60	55	2189	13	268	23						9				269
29	bi	7	14	25	6	32	132	7		14										
30	cv	15	19	13	11	25	471	10	2	19						2		1		
31	kh	5	40	66	28	65	0	3		40						2				
32	cm	1	17	19	54	17	108			19								1		
33	ca	11	4	1	6	1	0			4										
34	ky	0	13	3	7	8	0			13										
35	cf	0	78	65	169	23	337			78										
36	td	1	2	28	40	53	278		1	2										
37	cl	19	13	34	17	42	17	8	3	13						7		1		
38	cn	8	16	30	21	46	0	5	1	16						2				
39	co	12	8	160	30	43	67	11		8						1				
40	km	6	6	11	42	52	44	3	3	6										

**Table 5:** Accessibility guidelines that were identified in the study.

Principle	Guideline	Success Criteria	Level	Total
Perceivable	1.1 Text Alternatives	1.1.1 Missing alternative text	A	1364
Perceivable	1.3 Adaptable	1.3.1 Info and Relationships	A	486
Perceivable	1.4 Distinguishable	1.4.3 Contrast (Minimum)	AA	4481
Perceivable	2.1 Keyboard Accessible	2.1.1 Keyboard	A	57
Operable	2.2 Enough Time	2.2.2 Pause, Stop, Hide	A	12
Operable	2.2 Enough Time	2.2.1 Timing Adjustable	A	39
Operable	2.4 Navigable	2.4.1 Bypass Blocks	A	5
Operable	2.4 Navigable	2.4.2 Page Titled	A	37
Operable	2.4 Navigable	2.4.4 Link Purpose (In Context)	A	782
Operable	2.4 Navigable	2.4.6 Headings and Labels	AA	582
Understandable	3.1 Readable	3.1.1 Language of Page	A	60
Understandable	3.3 Input Assistance	3.3.2 Labels or Instructions	A	18
Robust	4.1 Compatible	4.1.2 Name, Role, Value	A	291

Where the first number refers to the accessibility principle, the second number relates to the guideline, and the third number to the success criteria defined by WCAG 2.1 [8].

## Data Availability

Data are available upon request from the corresponding author.

## References

[1] World Health Organization (WHO) World Report on Disability 2011. https://apps.who.int/iris/handle/10665/44575.

[2] Gorgenyi-Hegyes E., Nathan R.J., Fekete-Farkas M. (2021). Workplace Health Promotion, Employee Wellbeing and Loyalty during Covid-19 Pandemic—Large Scale Empirical Evidence from Hungary. Economies.

[3] Dragioti E., Li H., Tsitsas G., Lee K.H., Choi J., Kim J., Choi Y.J., Tsamakis K., Estradé A., Agorastos A. (2022). A large-scale meta-analytic atlas of mental health problems prevalence during the COVID-19 early pandemic. J. Med. Virol..

[4] Yu S.Y. (2021). A review of the accessibility of ACT COVID-19 information portals. Technol. Soc..

[5] Johns Hopkins University & Medicine COVID-19 Dashboard by the Center for Systems Science and Engineering (CSSE) at Johns Hopkins University (JHU). 1 September 2022. https://coronavirus.jhu.edu/map.html.

[6] Acosta-Vargas P., Salvador-Acosta B., Salvador-Ullauri L., Jadán-Guerrero J. (2022). Accessibility challenges of e-commerce websites. Peer J. Comput. Sci..

[7] World Health Organization (WHO) (2021). Blindness and Vision Impairment. https://www.who.int/news-room/fact-sheets/detail/blindness-and-visual-impairment.

[8] World Wide Web Consortium (2018). Web Content Accessibility Guidelines (WCAG) 2.1. https://www.w3.org/TR/WCAG21/.

[9] Preiser W., Smith K. (2011). Universal Design Handbook.

[10] World Wide Web Consortium (W3C) (2014). Website Accessibility Conformance Evaluation Methodology (WCAG-EM) 1.0. https://wave.webaim.org/.

[11] WebAIM (2021). WAVE Web Accessibility Evaluation Tool. https://wave.webaim.org/.

[12] Alismail S., Chipidza W. (2021). Accessibility Evaluation of COVID-19 Vaccine Registration Websites across the United States. J. Am. Med. Inform. Assoc..

[13] Howe J.L., Young C.R., Parau C.A., Trafton J.G., Ratwani R.M. (2021). Accessibility and usability of state health department COVID-19 vaccine websites: A qualitative study. JAMA Netw. Open.

[14] Fernández-Díaz E., Iglesias-Sánchez P.P., Jambrino-Maldonado C. (2020). Exploring WHO Communication during the COVID-19 Pandemic through the WHO Website Based on W3C Guidelines: Accessible for All?. Int. J. Environ. Res. Public Health.

[15] Acosta-Vargas P., Vergara C.F., Mendizabal R.P., Rodriguez M.A., Calvopina M., Kyriakidis N.C., Salvador-Acosta B., Prado E.O. (2022). Challenges in achieving accessibility on official COVID-19 websites. Hum. Factors Syst. Interact..

[16] Lorenzini M.-C., Wittich W. (2020). Factors related to the use of magnifying low vision aids: A scoping review. Disabil. Rehabil..

[17] Salvador-Ullauri L., Acosta-Vargas P., Gonzalez M., Luján-Mora S. (2020). Combined method for evaluating accessibility in serious games. Appl. Sci..

[18] W3C (2022). Accessibility Principles. Web Accessibility Initiative (WAI). W3C. https://www.w3.org/WAI/fundamentals/accessibility-principles/.

[19] W3C (2022). Web Content Accessibility Guidelines 1.0. https://www.w3.org/TR/WAI-WEBCONTENT/#priorities.

[20] W3C (2020). Web Content Accessibility Guidelines (WCAG) 2.0. https://www.w3.org/TR/2008/REC-WCAG20-20081211/.

[21] World Wide Web Consortium (2020). Web Content Accessibility Guidelines (WCAG) 2.2. https://www.w3.org/TR/WCAG22/.

[22] W3C (2021). W3C Accessibility Guidelines (WCAG) 3.0. https://www.w3.org/TR/wcag-3.0/.

[23] World Health Organization (WHO) (2017). Ten Facts on Disability. https://www.who.int/news-room/facts-in-pictures/detail/disabilities.

[24] GFMER (2021). Geneva Foundation for Medical Education and Research. www.gfmer.ch/Books/Reproductive_health/Human_sexual_differentiation.html.

[25] Acosta-Vargas P., Salvador-Acosta B., Calvopina M., Kyriakidis N., Ortiz-Prado E., Salvador-Ullauri L. (Dataset) Web Accessibility COVID-19 Website. Mendeley Data. 2022, V1. https://data.mendeley.com/datasets/9pkxzpcsm7/1.

[26] Shakeel T., Habib S., Boulila W., Koubaa A., Javed A.R., Rizwan M., Gadekallu T.R., Sufiyan M. (2022). A survey on COVID-19 impact in the healthcare domain: Worldwide market implementation, applications, security and privacy issues, challenges and future prospects. Complex Intell. Syst..

